# A New Approach for the Diagnosis of Myelodysplastic Syndrome Subtypes Based on Protein Interaction Analysis

**DOI:** 10.1038/s41598-019-49084-2

**Published:** 2019-09-02

**Authors:** Leona Chrastinová, Ondřej Pastva, Markéta Bocková, Nicholas S. Lynn, Pavel Šácha, Martin Hubálek, Jiří Suttnar, Roman Kotlín, Jana Štikarová, Alžběta Hlaváčková, Kristýna Pimková, Jaroslav Čermák, Jiří Homola, Jan E. Dyr

**Affiliations:** 1 Institute of Hematology and Blood Transfusion, Prague, Czech Republic; 2 Institute of Photonics and Electronics, Czech Academy of Sciences, Prague, Czech Republic; 3 Institute of Organic Chemistry and Biochemistry, Czech Academy of Sciences, Prague, Czech Republic

**Keywords:** Surface plasmon resonance, Myelodysplastic syndrome

## Abstract

Myelodysplastic syndromes (MDS) are a heterogeneous group of hematological malignancies with a high risk of transformation to acute myeloid leukemia (AML). MDS are associated with posttranslational modifications of proteins and variations in the protein expression levels. In this work, we present a novel interactomic diagnostic method based on both protein array and surface plasmon resonance biosensor technology, which enables monitoring of protein-protein interactions in a label-free manner. In contrast to conventional methods based on the detection of individual biomarkers, our presented method relies on measuring interactions between arrays of selected proteins and patient plasma. We apply this method to plasma samples obtained from MDS and AML patients, as well as healthy donors, and demonstrate that even a small protein array comprising six selected proteins allows the method to discriminate among different MDS subtypes and healthy donors.

## Introduction

Myelodysplastic syndromes (MDS) are a heterogeneous group of hematological malignancies that affect pluripotent hematopoietic stem cells^1^. It has been reported that about 30 percent of patients diagnosed with MDS progress to acute myeloid leukemia (AML)^2^. MDS are associated with various protein posttranslational modifications, different when compared with protein modifications in the normal population^3^. Moreover, changes in protein concentrations have been observed and several mutations have also been identified^4^. Methods targeting selected proteins thus present a promising avenue to detect the onset, and monitor the progression, of MDS.

Protein (micro)arrays enable the investigation of two protein attributes that can be altered due to disease: protein concentrations (quantitative proteomics), and interactions of proteins with other biomolecules (functional proteomics)^5,6^. Protein (micro)array-based techniques currently include sandwich immunoassays^7^, antigen capture immunoassays, and direct immunoassays^8^, whereas methods for data acquisition (readout methods) include fluorescence resonance energy transfer^9^, fluorescence correlation spectroscopy^10^, quartz crystal microbalance^11^, and mass spectrometry^12^. Protein (micro)array-based techniques that require protein labeling (with fluorescent or radioactive molecules) are highly sensitive; however, they suffer from adverse effects associated with the molecular labels, which affect protein activity and their ability to interact with antibodies or other proteins^6,13^. Therefore, the development of label-free methods has received a great deal of attention.

Surface plasmon resonance (SPR) is an optical method that enables monitoring of biomolecular interactions and quantification of biomolecules in a label-free manner^14^. Since their introduction in the 1990s, numerous SPR biosensor platforms have been developed for use in a variety of biomolecular applications; among these, SPR imaging (SPRi) biosensors allow for the parallelized observation of biomolecular interactions, increasing the throughput of SPR biosensor technology^15^. In the last decade, SPR biosensor technologies have been increasingly applied to the detection of biomolecules related to medical diagnosis^16^.

Although these current diagnostic methods to monitor specific protein concentrations represent a powerful and useful technology (both label-based and label-free), they require prior knowledge of the proteins being targeted (i.e., the selection of appropriate biorecognition elements, typically antibodies, for protein recognition and capture), which hampers the applicability of these methods for the diagnosis of complex diseases. This is especially problematic for diseases (such as oncohematological diseases) having molecular bases that are not fully understood.

Advances in the development of high-throughput screening technologies have created new opportunities for systematically investigating the complex networks of biomolecular interactions that constitutes the basis of diseases^17^. Giorgini and coworkers mapped interactions of Huntingtin (*Htt*), a protein whose aggregation plays a role in the beginning of Huntington’s disease, and identified *Htt*-protein interactions that mediates the aggregation process^18^. In another pair of studies, Srinivasa Rao *et al*. constructed a network of protein-protein interactions for Alzheimer’s disease employing a computational approach^19^, and Drusbosky *et al*. demonstrated that a computational approach allows for the mapping of the MDS mutanome^20^. Nevertheless, an investigation of protein interactions has not yet been used to diagnose diseases or their progression.

In this work we apply a novel interactomic diagnostic method of oncohematological diseases, which is based on both protein (micro)array and surface plasmon resonance biosensor technologies. In contrast to conventional methods based on the detection of biomarkers, the method proposed herein relies on measuring the interactions between selected proteins and patient plasma (without specifically targeting any distinct biomolecules). Therefore, the method does not require any previous knowledge of specific interactions (protein/protein or protein/antibody). We apply this method for the analysis of blood plasma samples obtained from MDS patients (three MDS subgroups, and MDS progressed into AML) and control samples from healthy donors. Interactions of proteins and plasma samples are observed and quantified in parallel using a multichannel SPR imaging biosensor without any protein labeling.

## Results

### Protein-protein interactions in MDS subgroups

Protein-protein interactions in MDS plasma were analyzed by a SPRi assay. This assay combines a protein chip, comprised of selected proteins having the potential to interact with blood plasma, with a SPR biosensor, which allows the observation of these interactions in a real-time and label-free manner. We monitored the interactions of blood plasma with a protein array containing six proteins (whose concentrations were found altered during MDS). Four proteins were selected based on literature data, where studies of MDS serum^21^ and proteomic studies of MDS plasma^22^ have revealed intracellular adhesion molecule 1 (ICAM), vascular cell adhesion protein 1 (VCAM), alpha-2-HS-glycoprotein (fetuin), and leucine-rich alpha-2-glycoprotein (LRG) as promising candidates. Two additional proteins - clusterin and S100A8 - were selected using the SPR/LC-MS/MS approach as discussed below.

**Figure 1:**
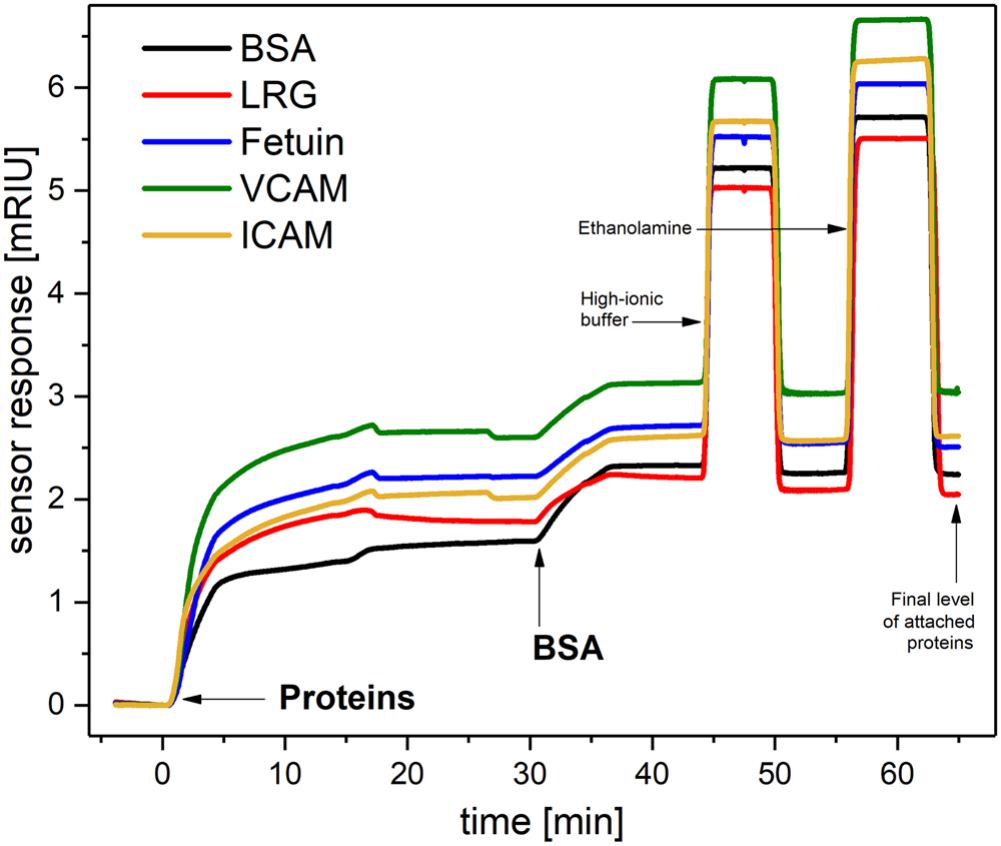
Sensorgram corresponding to the immobilization of selected proteins. Immobilization of proteins was performed in SA_4_-MgCl_2_ (ICAM, VCAM), or SA_5_ (fetuin, LRG), the concentration of all proteins was - 4 μg/ml. The sensor surface was then subsequently incubated with 5 μg/ml BSA in SA_5,_ with a high ionic strength PBNa buffer, and finally with 1 M EA to deactivate sensor surface. In the reference channel, the surface was functionalized only with covalently attached BSA.

**Figure 2:**
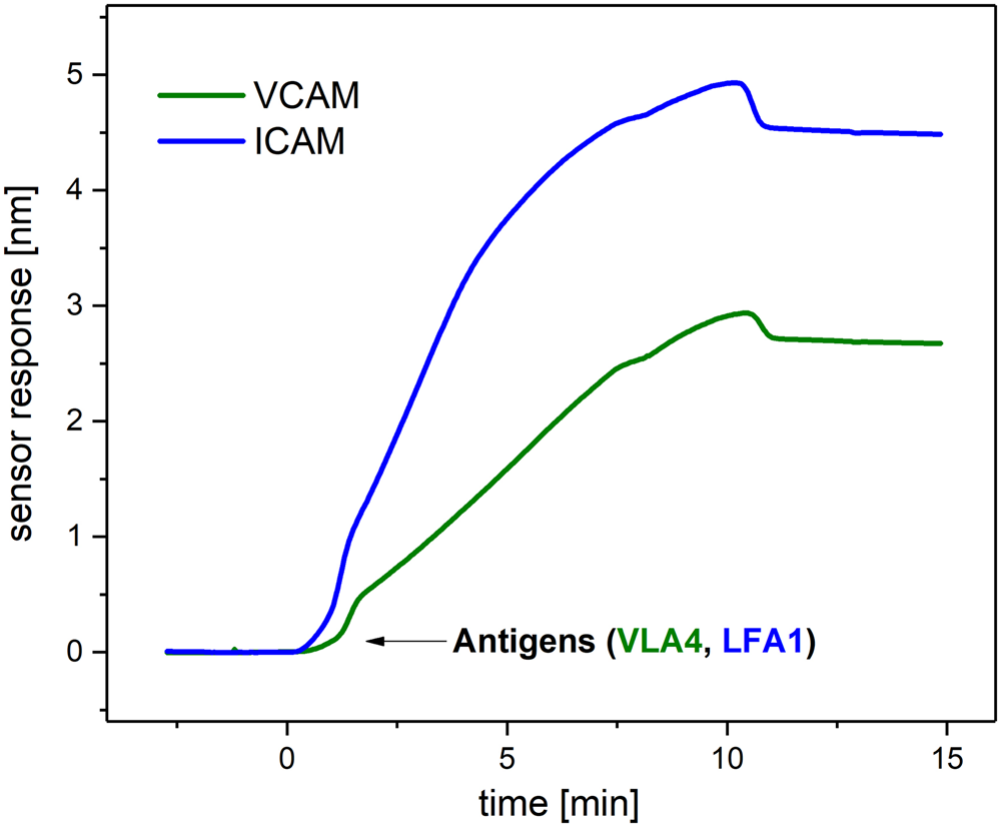
Typical sensor response to VLA4 interacting with VCAM and to LFA1 interacting with ICAM. VLA4 or LFA1 (concentration 4 μg/ml) in SA_4_-MgCl_2_ buffer were injected on the chip with immobilized VCAM or ICAM (concentration 4 μg/ml) in the respective channels, followed by an injection of running buffer (SA_4_-MgCl_2_).

In the first step of SPRi experiments, we immobilized six selected proteins onto the sensor chip surface. The sensorgram shown in Fig. 1 depicts the sensor response to the immobilization of MDS-related proteins in four sensing channels along with a non-reactive protein (bovine serum albumin - BSA) in a reference channel. The final level of the immobilized proteins was determined from the sensor response in buffer 5 min after the sensor surface was treated with ethanolamine hydrochloride (EA). Figure S1 in the Supplementary Information shows SPR sensorgrams for the immobilization of five different proteins on five different spots of the SPR chip. The reproducibility of immobilization was found to be better than 90% for each protein (obtained from four different chips). In independent SPR experiments we verified that the immobilization did not change the interaction properties for proteins known to act as a receptor for a specific antigens (VCAM is a receptor for VLA4, and ICAM is a receptor LFA1). In these experiments (using a 4-channel SPR biosensor), we flowed antigen molecules (VLA4 or LFA1) in buffer across the surface of a SPR chip having immobilized receptors (VCAM or ICAM, respectively) and monitored their interaction (Fig. 2). These results indicate that the immobilized receptors were able to bind their interacting partners and their function was not hampered after immobilization.

In the second step of SPRi experiments we measured interactions of immobilized proteins with plasma samples. The blood plasma samples were obtained from four different groups of MDS patients (divided according to their diagnosis) as well as from healthy controls (Table 1). These included MDS patients with refractory anemia (RA), refractory anemia with ringed sideroblasts (RARS), refractory cytopenia with multilineage dysplasia (RCMD), refractory anemia with excess blasts (RAEB), and patients who have progressed to acute myeloid leukemia (AML).

**Table 1:** Information on the patients and healthy controls involved in the study.

Diagnoses	Median Age (Age range)	Male/Female	Number of patients
RA, RARS	66 (30–94)	7/7	14
RCMD	53 (24–76)	8/8	16
RAEB	68 (51–77)	10/4	14
AML	66 (58–71)	5/6	11
Healthy control	48 (20–66)	15/11	26

RA - refractory anemia, RARS - refractory anemia with ringed sideroblasts, RCMD - refractory cytopenia with multilineage dysplasia, RAEB-I and II - refractory anemia with excess blasts subtype I and subtype II. The RAEB group includes 5 patients with RAEB-I (5–9% blasts) and 9 patients with RAEB-II (10–19% blasts). The RA, RARS group includes 4 patients with RA and 10 patients with RARS. AML patients are the patients who have been documented to progress from MDS to AML.

**Figure 3:**
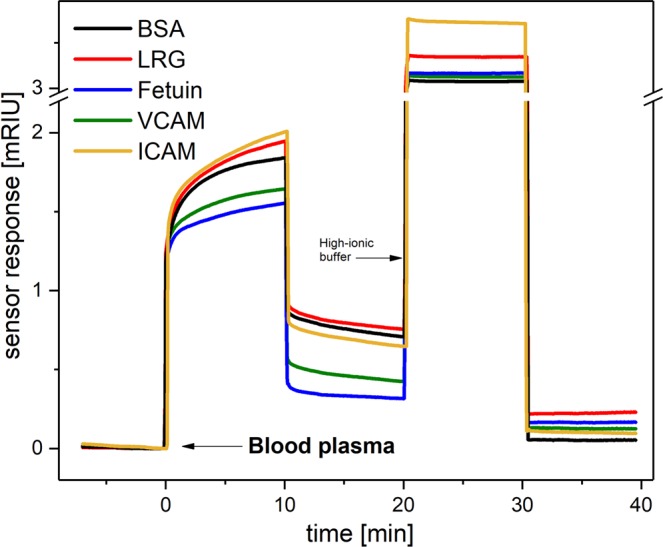
Typical sensor response to a blood plasma sample interacting with 5 different proteins. An initial injection of blood plasma (diluted to 1:9 with PBS_BSA_) was followed by an injection of PBS_BSA_, and then a high ionic strength PBNa, and finally followed by the PBS_BSA_ running buffer.

The sensorgram shown in Fig. 3 presents the sensor response to interactions between the five different immobilized proteins and blood plasma sample for a selected MDS patient. The final level of interacting plasma proteins was determined from the sensor response in buffer 10 min after the sensor surface was treated with high-ionic buffer. Additional raw data are shown in Fig. S2 in the Supplementary Information, which shows SPR sensorgrams corresponding to the interaction of plasma from three patients and two healthy controls, using four MDS-related proteins and BSA immobilized on the SPR chip. The SPRi biosensor used in these experiments exhibited a noise with a standard deviation of 3 × 10^−7^ RIU (calculated from the sensor response baseline). Given that the sensor response to the blood plasma samples was typically higher than 5 × 10^−5^ RIU, these sensor responses are rather robust, with a signal-to-noise ratio greater than 100. We also characterized the chip-to-chip reproducibility using four independent chips for all selected immobilized proteins, and found it to be around 85%.

**Figure 4:**
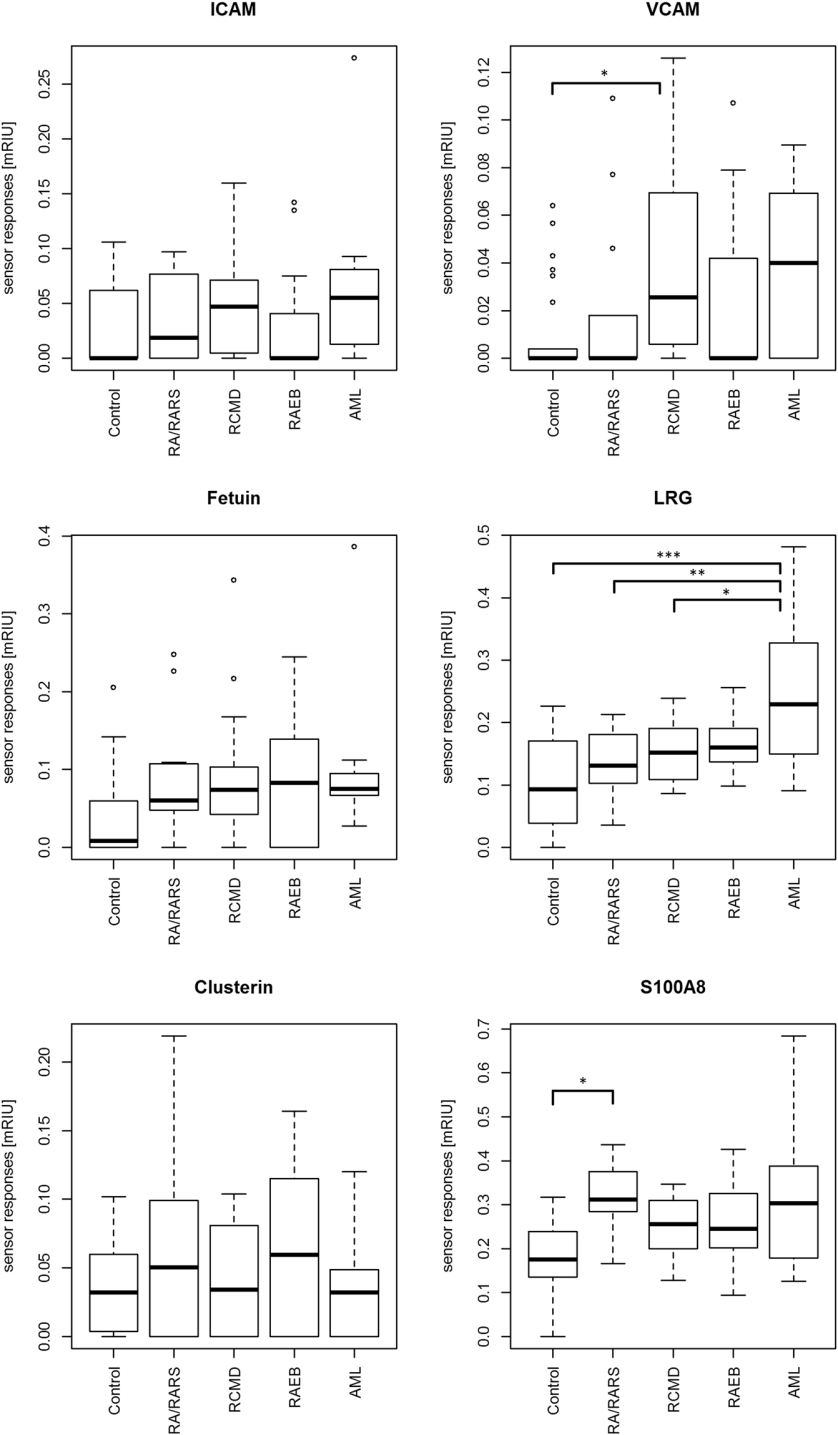
Box plots of SPR sensor responses. SPR sensor responses [mRIU] for plasma samples of MDS subgroups (RA/RARS, RCMD, RAEB, AML) and controls with selected immobilized proteins (ICAM, VCAM, fetuin, LRG, clusterin, S100A8). *P < 0.05, **P < 0.01, ***P < 0.001

The reference-compensated sensor responses are shown in Fig. 4. To process the interaction data, we first normalized the sensor responses to the interacting proteins to the respective level of the immobilized proteins. Then, in order to suppress the effect of various interferences (bulk refractive index variations, temperature fluctuations, etc.), we normalized the sensor responses to the reference-compensated sensor responses by subtracting the response in the reference channel (with immobilized BSA) from that in the sensing channels (with the immobilized MDS-related proteins).

Using a Shapiro normality test, we analyzed the normality of the reference-compensated data obtained for plasma from both MDS and AML patients and healthy controls, and we found that the data obtained for VCAM, ICAM, and fetuin were not normally distributed. Therefore, we used nonparametric tests (Kruskal-Wallis rank sum & pairwise comparisons using Nemenyi test) for their statistical evaluation. We did not reject the normal distribution of the data obtained from the interactions of LRG, clusterin, and S100A8.

We observed significant differences between the analyzed groups for VCAM (Kruskal-Wallis, P = 0.0225). Using a simultaneous post hoc test (Nemenyi test) we found that responses of SPR sensors with immobilized VCAM were significantly higher in the RCMD group with respect to controls (P = 0.04) (Fig. 4).

Significant differences were also observed between the groups analyzed for LRG (ANOVA, P = 0.00002) and for S100A8 (ANOVA, P = 0.0297). Using simultaneous tests for general linear hypotheses (Tukey multiple contrasts test) we found that sensor responses were significantly higher in the AML group with respect to controls for LRG (P < 0.001), and also in the RA/RARS group with respect to controls for S100A8 (P = 0.0341). Moreover, sensor responses were higher in the AML group with respect to RA/RARS, RCMD, and RAEB for immobilized LRG (P = 0.00525, P = 0.01943 and P = 0.07008, respectively) (Fig. 4).

**Figure 5:**
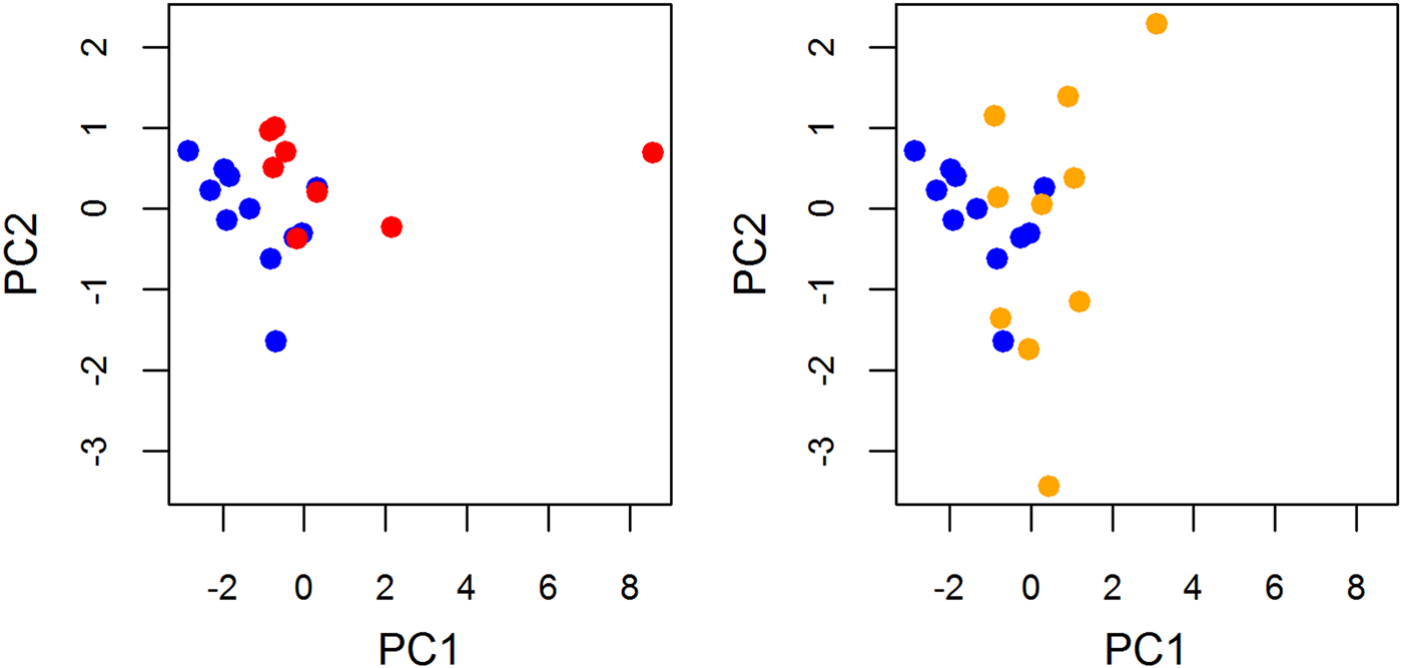
Principal component analysis of SPR sensor responses. PCA of SPR sensor responses [mRIU] performed on plasma samples of MDS subgroups (RA/RARS - yellow, n = 10), AML (red, n = 8), and controls (blue, n = 11), where SPR sensor responses were measured for all immobilized proteins (ICAM, VCAM, fetuin, LRG, clusterin, S100A8).

Analysis of the principal components of SPR sensor responses was performed on a subset of plasma samples of MDS subgroups, AML, and controls, where SPR sensor responses were measured for all immobilized proteins (ICAM, VCAM, fetuin, LRG, clusterin, S100A8). The results show separation of the analyzed groups: AML and controls, RA/RARS and controls, four variables (fetuin, LRG, clusterin and S100A8) contributed to the both components (Fig. 5).

### Mass spectrometry analysis

We observed significant differences in SPR sensor responses for immobilized VCAM or LRG among the analyzed groups. A four-channel spectroscopic SPR biosensor was used to capture interacting proteins from the plasma samples, and these interacting proteins were eluted for LC-MS/MS analysis. Proteins in both the patients and control plasma that interacted with the VCAM or LRG coated chips were identified using LC-MS/MS. At least two unique peptides were necessary to successfully identify a protein. Lists of the proteins identified in the plasma from two RAEB patients and one healthy control captured by LRG and VCAM proteins immobilized on the SPR chip, provided in Supplementary Information (Tables S1–S6), serve as an illustration of typical raw MS data. In contrast to healthy donor plasma, we identified the following proteins in MDS plasma: clusterin, protein S100A8, hornerin, serum amyloid A-1 protein, annexin A2, zinc-alpha-2-glycoprotein, gamma-glutamylcyclotransferase, ubiquitin-40S ribosomal protein S27a, etc. (Tables 2 and 3). Based on these findings and existing literature data about identified proteins and oncohematological diseases^23,24^, we subsequently chose two protein candidates, clusterin and S100A8, and analyzed a subset of plasma samples with the SPRi method.

**Table 2:** LC-MS/MS identification of proteins in MDS plasma interacting with immobilized VCAM.

Identified protein	AC	mass [Da]	Peptides	SC [%]
Annexin A2	P07355	38 604	3	13
Apolipoprotein A-I	P02647	30 778	52	72
Apolipoprotein C-II	P02655	11 284	3	29
Clusterin	P10909	52 495	4	14
Hornerin	Q86YZ3	282 390	9	15
Protein S100-A8	P05109	10 835	2	24
Serum amyloid A-1 protein	P0DJI8	13 532	20	54
Ubiquitin-40S ribosomal protein S27a	P62979	17 965	4	38
Zinc-alpha-2-glycoprotein	P25311	34 259	5	10

AC - accession number of protein in Swiss-prot database.

Peptides - number of unique peptides.

SC - protein sequence coverage in %.

**Table 3:** LC-MS/MS identification of proteins in MDS plasma interacting with immobilized LRG.

Identified protein	AC	mass [Da]	Peptides	SC [%]
Actin, cytoplasmic	P60709	41 737	6	20
Annexin A2	P07355	38 604	8	29
Gamma-glutamylcyclotransferase	O75223	21 008	2	13
Hornerin	Q86YZ3	282 390	22	16
Protein S100-A8	P05109	10 835	2	24
Suprabasin	Q6UWP8	60 541	4	19
Zinc-alpha-2-glycoprotein	P25311	34 259	7	32

AC - accession number of protein in Swiss-prot database.

Peptides - number of unique peptides.

SC - protein sequence coverage in %.

## Discussion

MDS diagnosis is a very complex process, complicated by poor reproducibility of morphologic analysis of bone marrow and peripheral blood^25^. The identification of novel plasmatic markers is desirable to make MDS diagnosis more accurate^26^. The existing blood protein biomarkers are not specific enough, while new biomarkers emerge very slowly^27^. Transition from single to multiple biomarkers, a signature that can provide higher diagnostic accuracy, is underway^28^. In this study we have designed a novel interactomic diagnostic method based on protein arrays. In contrast to conventional methods, this method relies on measuring of the interactions between selected proteins (known to be connected with the disease) and patient plasma - looking for the complex interactome and not only for the already known biomarkers.

The classification and risk assessment of myeloid neoplasms (AML and MDS) are guided by molecular genetic abnormalities. We attempted to use protein-protein interactions monitored by SPRi to extend genomic results to protein levels and to reveal other manifestations of the disease. In a pilot study we employed six target proteins: VCAM, ICAM, fetuin, clusterin, LRG, and S100A8.

The results of PCA analysis of the SPR sensor responses applied on a small subset of MDS and AML patients and controls revealed a clear separation of the analyzed groups: AML and controls, and RA/RARS and controls. Further SPR experiments showed that SPR sensor responses - obtained as a result of interactions between patient plasma proteins and immobilized LRG - were higher with respect to the controls. Moreover, in AML plasma the SPR sensor responses with LRG were significantly higher with respect to the RA/RARS, RCMD, and RAEB subgroups of MDS. Based on our results, LRG seems to be a suitable protein for SPR monitoring of MDS progression. These finding are in agreement with the published literature. Wen S. Y. *et al*. have proposed LRG as an independent prognostic factor for endometrial carcinoma^29^. LRG is a protein involved in neutrophil differentiation with unregulated expression, and has been classified as a marker of granulocytic differentiation^30^. Cavalcante *et al*. have identified LRG as a potential biomarker for diagnosis of B-cell acute lymphoblastic leukemia using lectin affinity chromatography and MS^31^.

We also found that SPR sensor responses of the RCMD group with respect to the controls for immobilized VCAM were significantly elevated; however, the sensor responses for protein interactions with VCAM and ICAM were around three times lower as compared with LRG. No differences in SPR sensor response were found among the studied MDS subgroups. These results agree with the findings by Sudhoff *et al*., who found non-significant differences in VCAM and ICAM levels between low-risk MDS (RA, RARS) and high-risk MDS (RAEB)^32^. On the other hand, Christiansen *et al*. observed the level of VCAM in serum reflects the disease progression as revealed by lymphocyte doubling time^33^. Serum levels of VCAM and ICAM were found to be higher in MDS patients when compared with the controls, and were related to MDS severity^21^.

We found non-significant differences in the SPR sensor responses between analyzed groups with immobilized fetuin, in spite of the fact that elevated fetuin levels have been linked with a higher risk of MDS, especially among overweight individuals^34^.

Due to both a lack of the other suitable MDS markers in existing literature as well as the statistically significant differences among groups studied for the immobilized LRG and VCAM, we analyzed proteins interacting with LRG and VCAM using LC-MS/MS. We identified changes of interacting proteins in MDS patient plasma related to the S100 gene: family protein S100A8 and hornerin. When compared to healthy donors, S100A8 is expressed more in the bone marrow mononuclear cells from MDS patients^23^. Results from LC-MS/MS identified additional interacting proteins, included clusterin, gamma-glutamyl cyclotransferase, suprabasin, annexin A2, alpha-1-acid glycoprotein, serum amyloid A-1 protein, ubiquitin-40S ribosomal protein S27a, actin cytoplasmic, apolipoprotein A-I, apolipoprotein C-II, and zinc-alpha-2-glycoprotein. Clusterin has previously been found to be overexpressed in AML patients^24^. Identification of proteins targeting the immobilized proteins on the sensor chip aids to reveal further suitable candidates for protein array-based SPRi experiments.

Despite findings that overexpression of clusterin is attributed to tumor formation, development, and metastasis^35^, and that silencing of clusterin can effectively induce apoptosis and inhibit the proliferation and invasion of HL-60 acute myeloid leukemia cells^24^, we did not find any significant differences of SPR sensor responses among the MDS subgroups for immobilized clusterin.

On the other hand, SPR sensor responses with immobilized S100A8 were significantly higher in RA/RARS plasma with respect to the controls. RA/RARS is characterized by anemia: the loss of red blood cells and ineffective erythropoiesis^36^. S100 proteins are involved in cell proliferation, differentiation, and death^37^ and have been already associated with MDS^38^. In particular, it has been reported that S100A8 negatively influences erythroid differentiation of hematopoietic stem cells and that genetic inactivation of S100A8 expression improved the erythroid differentiation^39^. The higher expression of S100A8 and S100A9 in bone marrow mononuclear cells from MDS patients compared to healthy donors has already been described^23^. The neutralization of S100A9 was reported to potentially improve the response of patients to erythropoietin^40^. The protein S100A8 is connected with erythropoiesis, and the RA/RARS subgroup is characterized by defects in erythroid development. Based on hitherto known data about S100A8 and our new results, S100A8 should be further tested as a suitable protein for SPR-based analysis of this MDS subgroup.

## Conclusions

We present a novel approach for the diagnosis of myelodysplastic syndromes based on the interactions between selected proteins and biomolecules in patient blood plasma. This interactomic method combines a protein chip, comprised of proteins that interact with blood plasma, and a surface plasmon resonance imaging biosensor, which allows the observation of these interactions in real-time and in a label-free manner. We applied this method to plasma samples obtained from MDS patients as well as healthy donors, and demonstrated that even when used with a small protein array (comprised of six selected proteins), it can discriminate among different MDS subgroups and healthy control donors. This method can be readily expanded and further improved, in terms of sensitivity and specificity, by employing a larger set of proteins: the SPRi biosensor technology used in this work is capable of analyzing hundreds of molecular interactions on a single chip in parallel. While this work demonstrates potential of this novel method for leukemias, the interatomic approach could also be adapted to a broad range of diseases that affect protein populations or interactions provided that suitable proteins can be identified for the SPR protein chip.

## Methods

### Chemicals and reagents

Recombinant Human Intracellular Adhesion Molecule 1 - ICAM, Recombinant Human Leukocyte Function Associated Antigen 1 – LFA1, Recombinant Human Vascular Cell Adhesion Protein 1 - VCAM, Recombinant Human Very Late Antigen 4 – VLA4, Recombinant Human Alpha-2-HS-Glycoprotein - fetuin, Recombinant Human Leucine-rich alpha-2-glycoprotein - LRG, Recombinant Human Clusterin, and Recombinant Human S100A8 were purchased from Novoprotein (Summit, NJ, USA).

16-Mercapto-hexa(ethylene glycol) hexadecanoid acid (HSC_11_(EG)_6_OCH_2_COOH) and 11-mercapto-tetra(ethyleneglycol)undecanol (HSC_11_(EG)_4_OH) were purchased from Prochimia (Gdansk, Poland). Dextran sulfate (DS), bovine serum albumin (BSA), sodium hydroxide (NaOH) and formic acid (FA) were purchased from Sigma-Aldrich (Prague, Czech Republic). Ethanolamine hydrochloride (EA), 1-hydroxypyrrolidine-2,5-dione (NHS) and 3-(ethyliminomethylideneamino)-N,N-dimethylpropan-1-amine (EDC) were purchased from Biacore (Uppsala, Sweden).

The buffers consisted of: PBS (0.01 M phosphate, 0,0027 M potassium chloride, 0.138 M sodium chloride, pH 7.4); PBNa (0.01 M phosphate, 0,009 M potassium chloride, 0.75 M sodium chloride, pH 7.4); SA_5_ (0.01 M sodium acetate, pH 5.0); SA_4_ (0.01 M sodium acetate, pH 4.0); SA_4_-MgCl_2_ (0.002 M MgCl_2_ in SA_4_); PBS_BSA_ (0.2% BSA in PBS (w/v)). All chemicals were of analytical grade. All buffers were prepared using deionized and double glass-distilled water on a Milli-Q50 (Millipore, Prague, Czech Republic).

### Blood plasma samples

Blood samples were collected both from patients with MDS diagnosed at the Institute of Hematology and Blood Transfusion, Prague, Czech Republic, and from healthy volunteers. The informed consent was obtained from all participants of this study at the time of blood collection. The study was approved by the Institute of Hematology and Blood Transfusion Ethics Committee, and all samples were obtained in accordance with the regulations of the ethical committee of the institute and with the Declaration of Helsinki. Human blood was drawn from patients and age- and sex- matched healthy donors by venipuncture into polypropylene tubes coated with EDTA. Plasma was obtained by centrifugation (5 min, 4000 × g) of blood samples. Plasma samples were stored at −70 °C until their analysis. Detailed information on the blood plasma samples used in this study is provided in Table 1.

### SPR biosensor platforms

Two SPR biosensor platforms developed at the Institute of Photonics and Electronics (Prague, Czech Republic) were used in this work. A high-resolution SPR imaging system with polarization contrast and internal referencing^41^ was used for the investigation of protein-protein interactions in MDS blood plasma samples. This SPRi system enables simultaneous analysis of biomolecular interactions in 5 × 5 individual flow-through sensing spots, by using a 5-channel flow-cell with two possible orientations of the flow chambers – horizontal and vertical. The first orientation of the flow-cell was used for surface functionalization, while the second (perpendicular) orientation was used for MDS plasma analysis. Passive mixing structures were employed in order to ensure a homogeneous sensor response along the flow chamber and to further improve sensing performance of the biosensor^42^. The response of the SPRi biosensor is expressed in refractive index units (RIU), which is related to the surface concentration of biomolecules on the sensor surface^41^.

A four-channel spectroscopic SPR biosensor was used to capture interacting proteins from the plasma of MDS patients for LC-MS/MS analysis (liquid chromatography combined with tandem mass spectrometry). In this SPR biosensor, the sensor response is expressed as a shift in the wavelength for which the resonance occurs, and is directly proportional to the mass of biomolecules attached to the surface of the sensor^15,43^.

Both SPR sensors were equipped with a temperature controller, and sample delivery to the sensor was carried out using a microfluidic flow cell in a near dispersionless manner^44^. We employed a reference channel in each experiment to compensate for the interfering effects. In the reference channel, the surface was functionalized only with covalently attached BSA.

### Immobilization of proteins on the SPR chip

In all experiments, immobilization of receptor proteins related to MDS was achieved via covalent attachment to a ω-carboxyalkylthiolate self-assembled monolayer (SAM). Details of both the preparation of the mixed SAM of HS-C_11_-(EG)_4_-OH and HS-C_11_-(EG)_6_-OCH_2_-COOH alkylthiols, as well as the *in situ* activation of carboxylic terminal groups, have been described previously^45,46^. After activation, the chip surface was incubated with SA_4_ (S100A8, clusterin), SA_4_-MgCl_2_ (ICAM, VCAM), or SA_5_ (fetuin, LRG). Immobilization of the respective receptor proteins to the activated surface was performed in SA_4_ (S100A8, clusterin), SA_4_-MgCl_2_ (ICAM, VCAM), or SA_5_ (fetuin, LRG) for 20 min (flow 5 µl/min, concentration of all proteins - 4 μg/ml). In order to increase the surface resistance to nonspecific adsorption, BSA was covalently attached, where the sensor surface was incubated with 5 μg/ml BSA in SA_5_ for 5 min (flow 20 µl/min). The high ionic strength PBNa buffer was injected for 5 min to remove any non-covalently bound receptor proteins or BSA. Finally, the sensor surface was treated with 1 M EA for 5 min to ensure deactivation of the carboxylic groups.

In order to confirm that the proteins maintained their interaction properties upon the immobilization, the proteins that are known to act as receptors for a specific antigen (VCAM and ICAM) were immobilized on the sensor chip. Respective antigens (VLA4 and LFA1, concentration - 4 μg/ml) in SA_4_-MgCl_2_ buffer were then flowed over the sensor chip for 10 min, after which the sensor surface was flushed with buffer (SA_4_-MgCl_2_).

### Detection of interacting proteins

After the immobilization of selected proteins we investigated their interaction with proteins in MDS plasma samples. Before we injected plasma sample, PBS_BSA_ buffer was flowed across the sensor surface until a stable baseline was reached. Then, plasma (diluted tenfold with PBS_BSA_ to achieve the best ratio between specific and unspecific sensor responses) was injected for 10 min, followed by an injection of PBS_BSA_. Finally, high ionic strength PBNa was flowed for 10 min, followed by the PBS_BSA_ running buffer.

### Mass spectrometry analysis

Interacting proteins were removed from the chip surface by 10 mM sodium hydroxide^47^. Mass spectrometry analysis was performed according to Tykvart *et al*.^48^. The samples were reduced with DTT, alkylated with iodoacetamide, and digested with trypsin for 10 h. Peptides were extracted and then dissolved in 0.1% formic acid (FA). The UltiMate 3000 RSLCnano system (Thermo Scientific) coupled to a TripleTOF 5600 mass spectrometer with a NanoSpray III source (Sciex) was used for sample analysis. Peptides were separated on an Acclaim PepMap100 analytical column (250 mm × 75 μm, 3 μm, Thermo Scientific). The buffer A was 0.1% FA, and the buffer B was 0.1% FA in acetonitrile. The concentration of buffer B was gradually increased from 5 to 30% over 95 min. Data were acquired over the range of m/z 350–1250 in MS/MS mode, the instrument acquired fragmentation spectra between 100–1600 m/z. The Protein Pilot 4.5 (AB Sciex) was used for protein identification against the UniProt *Homo sapiens* database (reviewed December, 2013)^48^.

### Statistical analysis

Statistical tests were used to examine the differences across all groups (MDS subgroups, controls). One-way ANOVA, Tukey multiple contrasts test, Kruskal-Wallis test, Nemenyi test, and principal component analysis were performed using R-software (R Core Team (2016). R: A language and environment for statistical computing. R Foundation for Statistical Computing, Vienna, Austria. URL https://www.R-project.org/). All tests for statistical significance were standardized at an alpha level of P < 0.05.

## Supplementary information

Supplementary information

## Data Availability

The data that support the findings of the current study are available from the corresponding author upon reasonable request.
