# Supplementary material for: A New Approach for the Diagnosis of Myelodysplastic Syndrome Subtypes Based on Protein Interaction Analysis
Source: Sci Rep. 2019 Sep 2;9:12647. doi: 10.1038/s41598-019-49084-2 (PMC6718656; doi:10.1038/s41598-019-49084-2)
Supplement: Supplementary file 1 — Supplementary information [file 41598_2019_49084_MOESM1_ESM.pdf]

Supplementary Information

A New Approach for the Diagnosis of Myelodysplastic Syndrome Subtypes Based on Protein Interaction Analysis

Leona Chrastinová, Ondřej Pastva, Markéta Bocková, Nicholas S. Lynn, Pavel Šácha, Martin Hubálek, Jiří Suttnar, Roman Kotlín, Jana Štikarová, Alžběta Hlaváčková, Kristýna Pimková, Jaroslav Čermák, Jiří Homola, Jan E. Dyr

Supplementary Figure S1. *Typical set of sensorgrams showing the immobilization of different proteins on a SPR chip. Each sensorgram corresponds to the immobilization of a single MDS-related protein.*

Supplementary Figure S2. *Typical set of sensorgrams showing the detection of protein-protein interactions on a SPR chip. Sensorgrams show the sensor response to the interactions of proteins from three different patients (and two healthy controls) using four different MDS-related proteins and BSA immobilized on the SPR chip.*

Supplementary Table S1. *List of identified proteins from SPR/LC-MS/MS experiment for MDS patient #1 with VCAM immobilized on the SPR sensor chip.*

Supplementary Table S2. *List of identified proteins from SPR/LC-MS/MS experiment for MDS patient #2 with VCAM immobilized on the SPR sensor chip.*

Supplementary Table S3. *List of identified proteins from SPR/LC-MS/MS experiment for MDS patient #1 with LRG immobilized on the SPR sensor chip.*

Supplementary Table S4. *List of identified proteins from SPR/LC-MS/MS experiment for MDS patient #2 with LRG immobilized on the SPR sensor chip.*

Supplementary Table S5. *List of identified proteins from SPR/LC-MS/MS experiment for the healthy control with VCAM immobilized on the SPR sensor chip.*

Supplementary Table S6. *List of identified proteins from SPR/LC-MS/MS experiment for the healthy control with LRG immobilized on the SPR sensor chip.*

Supplementary Figure S1

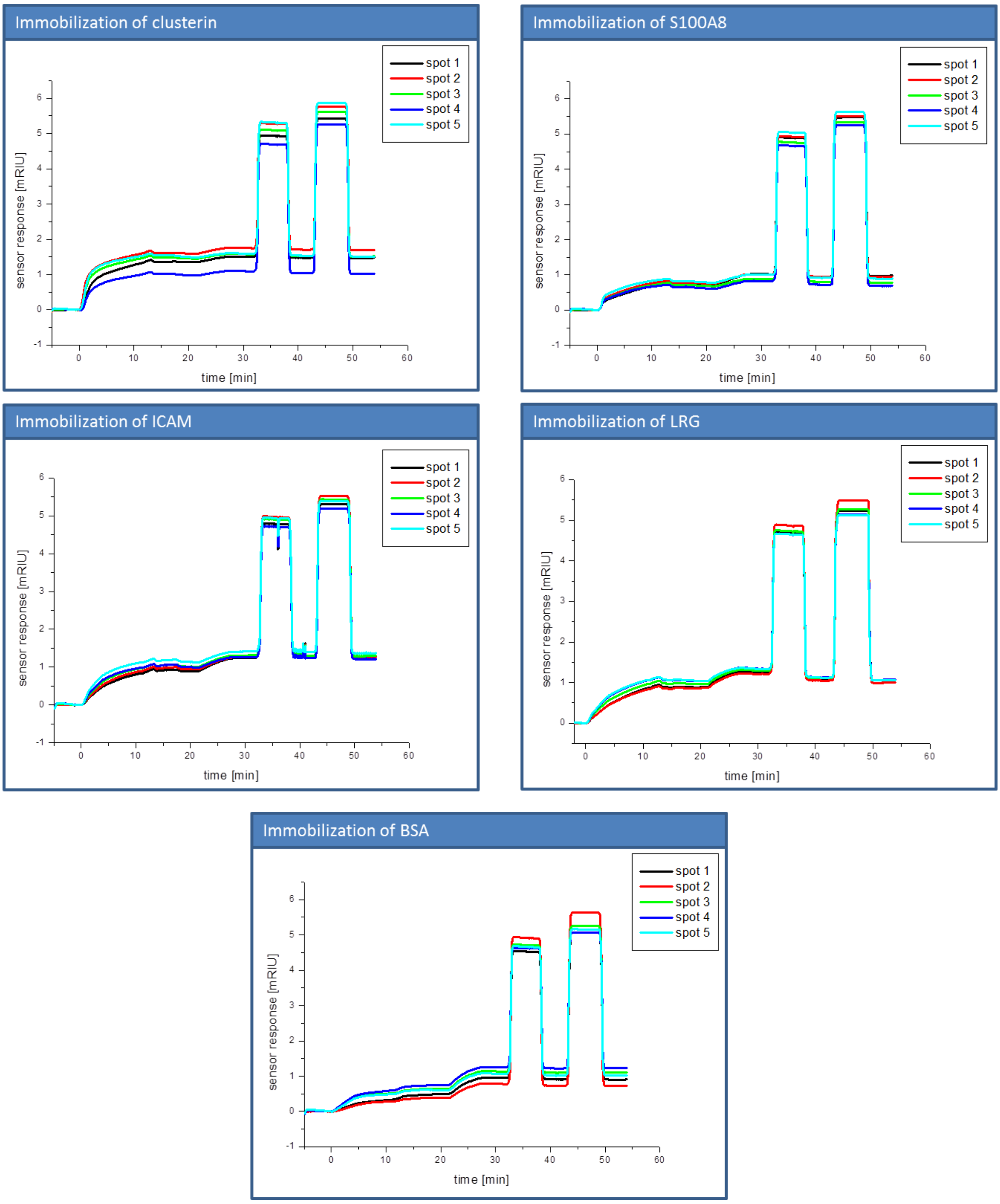

Supplementary Figure S2

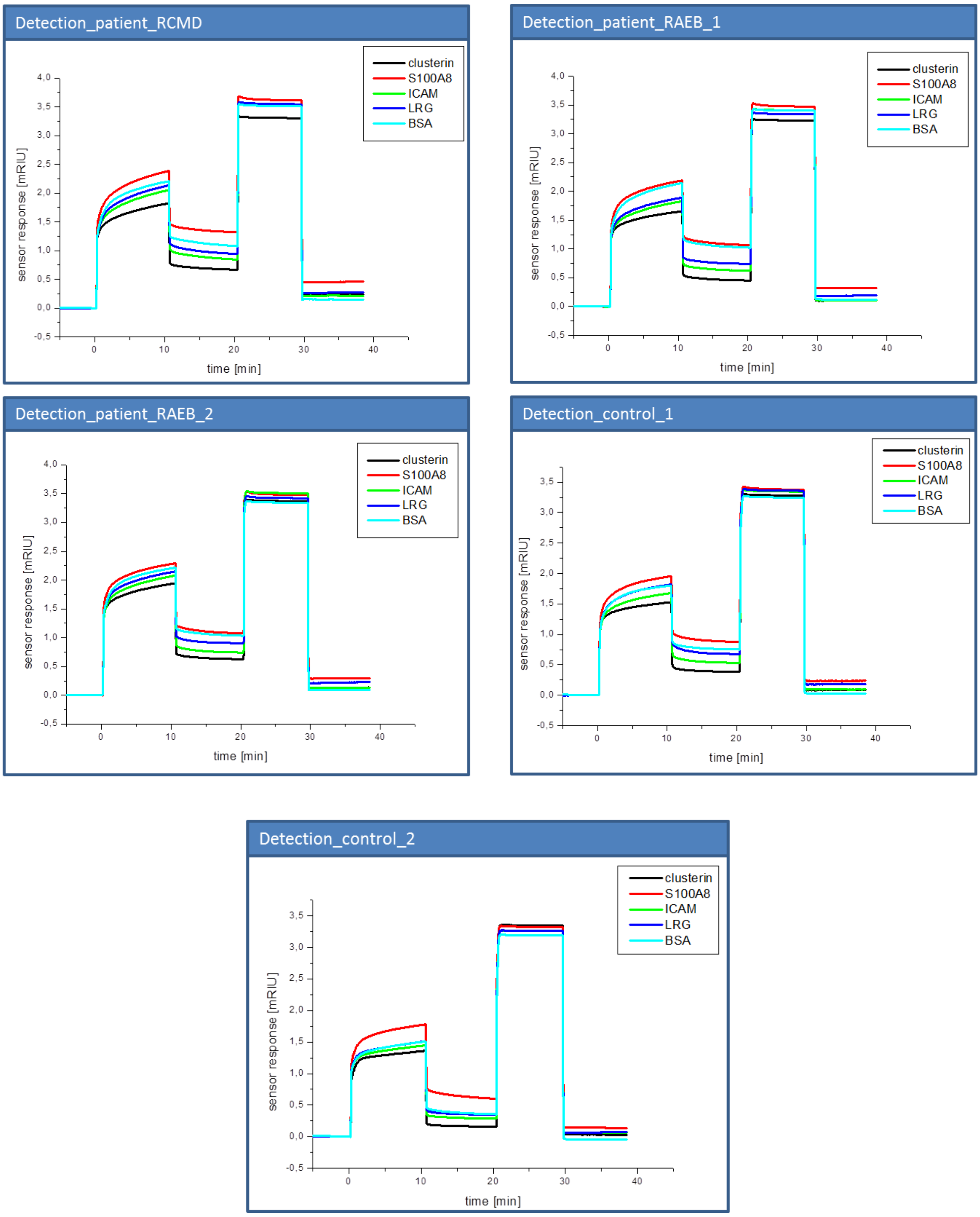

Supplementary Table S1.

Accession	Name	Peptides(95%)	%Cov(95)
sp|P02768|ALBU_HUMAN	Serum albumin OS=Homo sapiens GN=ALB PE=1 SV=2	16	32.18
sp|Q02413|DSG1_HUMAN	Desmoglein-1 OS=Homo sapiens GN=DSG1 PE=1 SV=2	11	14.97
sp|Q86YZ3|HORN_HUMAN	Hornerin OS=Homo sapiens GN=HRNR PE=1 SV=2	9	15.05
sp|P81605|DCD_HUMAN	Dermcidin OS=Homo sapiens GN=DCD PE=1 SV=2	8	56.36
sp|P14923|PLAK_HUMAN	Junction plakoglobin OS=Homo sapiens GN=JUP PE=1 SV=3	8	17.72
sp|P15924|DESP_HUMAN	Desmoplakin OS=Homo sapiens GN=DSP PE=1 SV=3	7	3.41
sp|Q5D862|FILA2_HUMAN	Filaggrin-2 OS=Homo sapiens GN=FLG2 PE=1 SV=1	7	5.10
sp|P31944|CASPE_HUMAN	Caspase-14 OS=Homo sapiens GN=CASP14 PE=1 SV=2	6	37.19
sp|P04406|G3P_HUMAN	Glyceraldehyde-3-phosphate dehydrogenase OS=Homo sapiens GN=GAPDH PE=1 SV=3	5	27.16
sp|P31151|S10A7_HUMAN	Protein S100-A7 OS=Homo sapiens GN=S100A7 PE=1 SV=4	5	50.50
sp|Q9NZT1|CALL5_HUMAN	Calmodulin-like protein 5 OS=Homo sapiens GN=CALML5 PE=1 SV=2	4	37.67
sp|Q08554|DSC1_HUMAN	Desmocollin-1 OS=Homo sapiens GN=DSC1 PE=1 SV=2	4	6.04
sp|P12273|PIP_HUMAN	Prolactin-inducible protein OS=Homo sapiens GN=PIP PE=1 SV=1	4	33.56
sp|P07339|CATD_HUMAN	Cathepsin D OS=Homo sapiens GN=CTSD PE=1 SV=1	3	9.47
sp|P06702|S10A9_HUMAN	Protein S100-A9 OS=Homo sapiens GN=S100A9 PE=1 SV=1	3	49.12
sp|P29508|SPB3_HUMAN	Serpin B3 OS=Homo sapiens GN=SERPINB3 PE=1 SV=2	3	7.69
sp|P48594|SPB4_HUMAN	Serpin B4 OS=Homo sapiens GN=SERPINB4 PE=1 SV=2	3	7.69
sp|P25311|ZA2G_HUMAN	Zinc-alpha-2-glycoprotein OS=Homo sapiens GN=AZGP1 PE=1 SV=2	3	16.44
sp|P05089|ARGI1_HUMAN	Arginase-1 OS=Homo sapiens GN=ARG1 PE=1 SV=2	2	11.49
sp|P04040|CATA_HUMAN	Catalase OS=Homo sapiens GN=CAT PE=1 SV=3	2	4.74
sp|Q15517|CDSN_HUMAN	Corneodesmosin OS=Homo sapiens GN=CDSN PE=1 SV=3	2	5.10
sp|Q01469|FABP5_HUMAN	Fatty acid-binding protein, epidermal OS=Homo sapiens GN=FABP5 PE=1 SV=3	2	18.52
sp|P01876|IGHA1_HUMAN	Ig alpha-1 chain C region OS=Homo sapiens GN=IGHA1 PE=1 SV=2	2	7.37
sp|P61626|LYSC_HUMAN	Lysozyme C OS=Homo sapiens GN=LYZ PE=1 SV=1	2	16.22
sp|Q06830|PRDX1_HUMAN	Peroxiredoxin-1 OS=Homo sapiens GN=PRDX1 PE=1 SV=1	2	11.56
sp|P32119|PRDX2_HUMAN	Peroxiredoxin-2 OS=Homo sapiens GN=PRDX2 PE=1 SV=5	2	9.60
sp|P05109|S10A8_HUMAN	Protein S100-A8 OS=Homo sapiens GN=S100A8 PE=1 SV=1	2	23.66
sp|Q08188|TGM3_HUMAN	Protein-glutamine gamma-glutamyltransferase E OS=Homo sapiens GN=TGM3 PE=1 SV=4	2	5.20
sp|Q96P63|SPB12_HUMAN	Serpin B12 OS=Homo sapiens GN=SERPINB12 PE=1 SV=1	2	7.65

Supplementary Table S2.

Accession	Name	Peptides(95%)	%Cov(95)
sp|P81605|DCD_HUMAN	Dermcidin OS=Homo sapiens GN=DCD PE=1 SV=2	28	56.36
sp|P19320|VCAM1_HUMAN	Vascular cell adhesion protein 1 OS=Homo sapiens GN=VCAM1 PE=1 SV=1	24	23.14
sp|P0DJI8|SAA1_HUMAN	Serum amyloid A-1 protein OS=Homo sapiens GN=SAA1 PE=1 SV=1	20	54.10
sp|Q9NZT1|CALL5_HUMAN	Calmodulin-like protein 5 OS=Homo sapiens GN=CALML5 PE=1 SV=2	15	53.42
sp|P02671|FIBA_HUMAN	Fibrinogen alpha chain OS=Homo sapiens GN=FGA PE=1 SV=2	15	18.59
sp|P02768|ALBU_HUMAN	Serum albumin OS=Homo sapiens GN=ALB PE=1 SV=2	14	18.23
sp|P0DJI9|SAA2_HUMAN	Serum amyloid A-2 protein OS=Homo sapiens GN=SAA2 PE=1 SV=1	13	50.82
sp|Q86YZ3|HORN_HUMAN	Hornerin OS=Homo sapiens GN=HRNR PE=1 SV=2	11	8.67
sp|P02656|APOC3_HUMAN	Apolipoprotein C-III OS=Homo sapiens GN=APOC3 PE=1 SV=1	8	37.37
sp|P15924|DESP_HUMAN	Desmoplakin OS=Homo sapiens GN=DSP PE=1 SV=3	6	3.34
sp|P02675|FIBB_HUMAN	Fibrinogen beta chain OS=Homo sapiens GN=FGB PE=1 SV=2	6	11.81
sp|P20930|FILA_HUMAN	Filaggrin OS=Homo sapiens GN=FLG PE=1 SV=3	6	3.64
sp|P06702|S10A9_HUMAN	Protein S100-A9 OS=Homo sapiens GN=S100A9 PE=1 SV=1	6	28.07
sp|P25311|ZA2G_HUMAN	Zinc-alpha-2-glycoprotein OS=Homo sapiens GN=AZGP1 PE=1 SV=2	5	10.40
sp|P04003|C4BPA_HUMAN	C4b-binding protein alpha chain OS=Homo sapiens GN=C4BPA PE=1 SV=2	4	4.35
sp|P10909|CLUS_HUMAN	Clusterin OS=Homo sapiens GN=CLU PE=1 SV=1	4	14.03
sp|P01876|IGHA1_HUMAN	Ig alpha-1 chain C region OS=Homo sapiens GN=IGHA1 PE=1 SV=2	4	11.90
sp|P0CG47|UBB_HUMAN	Polyubiquitin-B OS=Homo sapiens GN=UBB PE=1 SV=1	4	77.29
sp|P0CG48|UBC_HUMAN	Polyubiquitin-C OS=Homo sapiens GN=UBC PE=1 SV=3	4	77.52
sp|P62979|RS27A_HUMAN	Ubiquitin-40S ribosomal protein S27a OS=Homo sapiens GN=RPS27A PE=1 SV=2	4	37.82
sp|P62987|RL40_HUMAN	Ubiquitin-60S ribosomal protein L40 OS=Homo sapiens GN=UBA52 PE=1 SV=2	4	46.09
sp|P02649|APOE_HUMAN	Apolipoprotein E OS=Homo sapiens GN=APOE PE=1 SV=1	3	11.99
sp|P01040|CYTA_HUMAN	Cystatin-A OS=Homo sapiens GN=CSTA PE=1 SV=1	3	33.67
sp|Q02413|DSG1_HUMAN	Desmoglein-1 OS=Homo sapiens GN=DSG1 PE=1 SV=2	3	4.67
sp|P01877|IGHA2_HUMAN	Ig alpha-2 chain C region OS=Homo sapiens GN=IGHA2 PE=1 SV=3	3	7.94
sp|P14923|PLAK_HUMAN	Junction plakoglobin OS=Homo sapiens GN=JUP PE=1 SV=3	3	6.71
sp|P60709|ACTB_HUMAN	Actin, cytoplasmic 1 OS=Homo sapiens GN=ACTB PE=1 SV=1	2	4.80
sp|P63261|ACTG_HUMAN	Actin, cytoplasmic 2 OS=Homo sapiens GN=ACTG1 PE=1 SV=1	2	4.80
sp|P01009|A1AT_HUMAN	Alpha-1-antitrypsin OS=Homo sapiens GN=SERPINA1 PE=1 SV=3	2	9.09
sp|P02652|APOA2_HUMAN	Apolipoprotein A-II OS=Homo sapiens GN=APOA2 PE=1 SV=1	2	33.00
sp|P02655|APOC2_HUMAN	Apolipoprotein C-II OS=Homo sapiens GN=APOC2 PE=1 SV=1	2	29.70
sp|P31944|CASPE_HUMAN	Caspase-14 OS=Homo sapiens GN=CASP14 PE=1 SV=2	2	14.88
sp|Q08554|DSC1_HUMAN	Desmocollin-1 OS=Homo sapiens GN=DSC1 PE=1 SV=2	2	3.24
sp|P02679|FIBG_HUMAN	Fibrinogen gamma chain OS=Homo sapiens GN=FGG PE=1 SV=3	2	5.30
sp|Q5D862|FILA2_HUMAN	Filaggrin-2 OS=Homo sapiens GN=FLG2 PE=1 SV=1	2	1.42
sp|P04206|KV307_HUMAN	Ig kappa chain V-III region GOL OS=Homo sapiens PE=1 SV=1	2	31.19
sp|P18135|KV312_HUMAN	Ig kappa chain V-III region HAH OS=Homo sapiens PE=2 SV=1	2	26.36
sp|P18136|KV313_HUMAN	Ig kappa chain V-III region HIC OS=Homo sapiens PE=2 SV=2	2	26.36
sp|P01620|KV302_HUMAN	Ig kappa chain V-III region SIE OS=Homo sapiens PE=1 SV=1	2	31.19
sp|P01622|KV304_HUMAN	Ig kappa chain V-III region Ti OS=Homo sapiens PE=1 SV=1	2	31.19
sp|P01623|KV305_HUMAN	Ig kappa chain V-III region WOL OS=Homo sapiens PE=1 SV=1	2	31.19
sp|P61626|LYSC_HUMAN	Lysozyme C OS=Homo sapiens GN=LYZ PE=1 SV=1	2	8.11
sp|P12273|PIP_HUMAN	Prolactin-inducible protein OS=Homo sapiens GN=PIP PE=1 SV=1	2	15.07
sp|P31151|S10A7_HUMAN	Protein S100-A7 OS=Homo sapiens GN=S100A7 PE=1 SV=4	2	12.87
sp|P02810|PRPC_HUMAN	Salivary acidic proline-rich phosphoprotein 1/2 OS=Homo sapiens GN=PRH1 PE=1 S	2	18.67
sp|P07225|PROS_HUMAN	Vitamin K-dependent protein S OS=Homo sapiens GN=PROS1 PE=1 SV=1	2	3.25
sp|P04004|VTNC_HUMAN	Vitronectin OS=Homo sapiens GN=VTN PE=1 SV=1	2	5.65

Supplementary Table S3.

Accession	Name	Peptides(95%)	%Cov(95)
sp|P15924|DESP_HUMAN	Desmoplakin OS=Homo sapiens GN=DSP PE=1 SV=3	40	15.99
sp|P81605|DCD_HUMAN	Dermcidin OS=Homo sapiens GN=DCD PE=1 SV=2	17	56.36
sp|Q86YZ3|HORN_HUMAN	Hornerin OS=Homo sapiens GN=HRNR PE=1 SV=2	17	14.70
sp|P14923|PLAK_HUMAN	Junction plakoglobin OS=Homo sapiens GN=JUP PE=1 SV=3	15	24.16
sp|P02768|ALBU_HUMAN	Serum albumin OS=Homo sapiens GN=ALB PE=1 SV=2	15	26.93
sp|Q02413|DSG1_HUMAN	Desmoglein-1 OS=Homo sapiens GN=DSG1 PE=1 SV=2	13	13.63
sp|P31944|CASPE_HUMAN	Caspase-14 OS=Homo sapiens GN=CASP14 PE=1 SV=2	12	45.04
sp|P07355|ANXA2_HUMAN	Annexin A2 OS=Homo sapiens GN=ANXA2 PE=1 SV=2	8	29.20
sp|Q13835|PKP1_HUMAN	Plakophilin-1 OS=Homo sapiens GN=PKP1 PE=1 SV=2	8	12.18
sp|P04406|G3P_HUMAN	Glyceraldehyde-3-phosphate dehydrogenase OS=Homo sapiens GN=GAPDH PE=1 SV=3	7	26.57
sp|P12273|PIP_HUMAN	Prolactin-inducible protein OS=Homo sapiens GN=PIP PE=1 SV=1	7	56.85
sp|Q08188|TGM3_HUMAN	Protein-glutamine gamma-glutamyltransferase E OS=Homo sapiens GN=TGM3 PE=1 SV=4	7	13.56
sp|P25311|ZA2G_HUMAN	Zinc-alpha-2-glycoprotein OS=Homo sapiens GN=AZGP1 PE=1 SV=2	7	31.54
sp|P60709|ACTB_HUMAN	Actin, cytoplasmic 1 OS=Homo sapiens GN=ACTB PE=1 SV=1	6	20.00
sp|P63261|ACTG_HUMAN	Actin, cytoplasmic 2 OS=Homo sapiens GN=ACTG1 PE=1 SV=1	6	20.00
sp|Q9NZT1|CALL5_HUMAN	Calmodulin-like protein 5 OS=Homo sapiens GN=CALML5 PE=1 SV=2	6	45.21
sp|P05089|ARGI1_HUMAN	Arginase-1 OS=Homo sapiens GN=ARG1 PE=1 SV=2	5	13.04
sp|P20930|FILA_HUMAN	Filaggrin OS=Homo sapiens GN=FLG PE=1 SV=3	5	2.09
sp|Q5D862|FILA2_HUMAN	Filaggrin-2 OS=Homo sapiens GN=FLG2 PE=1 SV=1	5	4.56
sp|P31151|S10A7_HUMAN	Protein S100-A7 OS=Homo sapiens GN=S100A7 PE=1 SV=4	5	43.56
sp|Q08554|DSC1_HUMAN	Desmocollin-1 OS=Homo sapiens GN=DSC1 PE=1 SV=2	4	5.82
sp|P47929|LEG7_HUMAN	Galectin-7 OS=Homo sapiens GN=LGALS7 PE=1 SV=2	4	36.03
sp|Q6UWP8|SBSN_HUMAN	Suprabasin OS=Homo sapiens GN=SBSN PE=2 SV=2	4	18.81
sp|Q06830|PRDX1_HUMAN	Peroxiredoxin-1 OS=Homo sapiens GN=PRDX1 PE=1 SV=1	3	15.08
sp|Q96P63|SPB12_HUMAN	Serpin B12 OS=Homo sapiens GN=SERPINB12 PE=1 SV=1	3	9.38
sp|P10599|THIO_HUMAN	Thioredoxin OS=Homo sapiens GN=TXN PE=1 SV=3	3	22.86
sp|Q15517|CDSN_HUMAN	Corneodesmosin OS=Homo sapiens GN=CDSN PE=1 SV=3	2	5.10
sp|O75223|GGCT_HUMAN	Gamma-glutamylcyclotransferase OS=Homo sapiens GN=GGCT PE=1 SV=1	2	12.77
sp|P04792|HSPB1_HUMAN	Heat shock protein beta-1 OS=Homo sapiens GN=HSPB1 PE=1 SV=2	2	9.76
sp|Q96DR8|MUCL1_HUMAN	Mucin-like protein 1 OS=Homo sapiens GN=MUCL1 PE=1 SV=1	2	10.00
sp|P32119|PRDX2_HUMAN	Peroxiredoxin-2 OS=Homo sapiens GN=PRDX2 PE=1 SV=5	2	10.61
sp|P0CG47|UBB_HUMAN	Polyubiquitin-B OS=Homo sapiens GN=UBB PE=1 SV=1	2	32.75
sp|P0CG48|UBC_HUMAN	Polyubiquitin-C OS=Homo sapiens GN=UBC PE=1 SV=3	2	32.85
sp|P06702|S10A9_HUMAN	Protein S100-A9 OS=Homo sapiens GN=S100A9 PE=1 SV=1	2	24.56
sp|P14618|KPYM_HUMAN	Pyruvate kinase PKM OS=Homo sapiens GN=PKM PE=1 SV=4	2	4.14
sp|O95969|SG1D2_HUMAN	Secretoglobin family 1D member 2 OS=Homo sapiens GN=SCGB1D2 PE=2 SV=1	2	22.22
sp|Q5T750|XP32_HUMAN	Skin-specific protein 32 OS=Homo sapiens GN=XP32 PE=1 SV=1	2	7.60
sp|P35326|SPR2A_HUMAN	Small proline-rich protein 2A OS=Homo sapiens GN=SPRR2A PE=1 SV=1	2	43.06
sp|P35325|SPR2B_HUMAN	Small proline-rich protein 2B OS=Homo sapiens GN=SPRR2B PE=2 SV=1	2	43.06
sp|P22532|SPR2D_HUMAN	Small proline-rich protein 2D OS=Homo sapiens GN=SPRR2D PE=2 SV=2	2	30.56
sp|P22531|SPR2E_HUMAN	Small proline-rich protein 2E OS=Homo sapiens GN=SPRR2E PE=2 SV=2	2	43.06
sp|Q9BYE4|SPR2G_HUMAN	Small proline-rich protein 2G OS=Homo sapiens GN=SPRR2G PE=2 SV=1	2	30.14
sp|P68363|TBA1B_HUMAN	Tubulin alpha-1B chain OS=Homo sapiens GN=TUBA1B PE=1 SV=1	2	5.10
sp|Q9BQE3|TBA1C_HUMAN	Tubulin alpha-1C chain OS=Homo sapiens GN=TUBA1C PE=1 SV=1	2	5.12
sp|P62979|RS27A_HUMAN	Ubiquitin-40S ribosomal protein S27a OS=Homo sapiens GN=RPS27A PE=1 SV=2	2	16.03
sp|P62987|RL40_HUMAN	Ubiquitin-60S ribosomal protein L40 OS=Homo sapiens GN=UBA52 PE=1 SV=2	2	19.53
sp|Q8WWY7|WFD12_HUMAN	WAP four-disulfide core domain protein 12 OS=Homo sapiens GN=WFDC12 PE=2 SV=1	2	23.42

Supplementary Table S4.

Accession	Name	Peptides(95%)	%Cov(95)
sp|P02768|ALBU_HUMAN	Serum albumin OS=Homo sapiens GN=ALB PE=1 SV=2	33	48.11
sp|P81605|DCD_HUMAN	Dermcidin OS=Homo sapiens GN=DCD PE=1 SV=2	24	56.36
sp|Q9NZT1|CALL5_HUMAN	Calmodulin-like protein 5 OS=Homo sapiens GN=CALML5 PE=1 SV=2	20	48.63
sp|P02656|APOC3_HUMAN	Apolipoprotein C-III OS=Homo sapiens GN=APOC3 PE=1 SV=1	14	56.57
sp|P15924|DESP_HUMAN	Desmoplakin OS=Homo sapiens GN=DSP PE=1 SV=3	13	5.75
sp|P14923|PLAK_HUMAN	Junction plakoglobin OS=Homo sapiens GN=JUP PE=1 SV=3	10	16.78
sp|P02765|FETUA_HUMAN	Alpha-2-HS-glycoprotein OS=Homo sapiens GN=AHSG PE=1 SV=1	7	30.25
sp|P10909|CLUS_HUMAN	Clusterin OS=Homo sapiens GN=CLU PE=1 SV=1	5	14.03
sp|Q02413|DSG1_HUMAN	Desmoglein-1 OS=Homo sapiens GN=DSG1 PE=1 SV=2	5	4.48
sp|P06702|S10A9_HUMAN	Protein S100-A9 OS=Homo sapiens GN=S100A9 PE=1 SV=1	5	24.56
sp|P07355|ANXA2_HUMAN	Annexin A2 OS=Homo sapiens GN=ANXA2 PE=1 SV=2	4	17.11
sp|P05089|ARGI1_HUMAN	Arginase-1 OS=Homo sapiens GN=ARG1 PE=1 SV=2	4	18.63
sp|P01834|IGKC_HUMAN	Ig kappa chain C region OS=Homo sapiens GN=IGKC PE=1 SV=1	4	48.11
sp|P02763|A1AG1_HUMAN	Alpha-1-acid glycoprotein 1 OS=Homo sapiens GN=ORM1 PE=1 SV=1	3	14.43
sp|Q5D862|FILA2_HUMAN	Filaggrin-2 OS=Homo sapiens GN=FLG2 PE=1 SV=1	3	0.96
sp|P04406|G3P_HUMAN	Glyceraldehyde-3-phosphate dehydrogenase OS=Homo sapiens GN=GAPDH PE=1 SV=3	3	12.84
sp|Q96P63|SPB12_HUMAN	Serpin B12 OS=Homo sapiens GN=SERPINB12 PE=1 SV=1	3	13.09
sp|P0DJI8|SAA1_HUMAN	Serum amyloid A-1 protein OS=Homo sapiens GN=SAA1 PE=1 SV=1	3	40.98
sp|P25311|ZA2G_HUMAN	Zinc-alpha-2-glycoprotein OS=Homo sapiens GN=AZGP1 PE=1 SV=2	3	14.43
sp|Q08554|DSC1_HUMAN	Desmocollin-1 OS=Homo sapiens GN=DSC1 PE=1 SV=2	2	3.24
sp|P20930|FILA_HUMAN	Filaggrin OS=Homo sapiens GN=FLG PE=1 SV=3	2	0.64
sp|P31943|HNRH1_HUMAN	Heterogeneous nuclear ribonucleoprotein H OS=Homo sapiens GN=HNRNPH1 PE=1 SV=4	2	9.13
sp|Q86YZ3|HORN_HUMAN	Hornerin OS=Homo sapiens GN=HRNR PE=1 SV=2	2	3.72
sp|P05109|S10A8_HUMAN	Protein S100-A8 OS=Homo sapiens GN=S100A8 PE=1 SV=1	2	23.66
sp|P35637|FUS_HUMAN	RNA-binding protein FUS OS=Homo sapiens GN=FUS PE=1 SV=1	2	10.65

Supplementary Table S5.

Accession	Name	Peptides(95%)	%Cov(95)
sp|P02768|ALBU_HUMAN	Serum albumin OS=Homo sapiens GN=ALB PE=1 SV=2	52	60.10
sp|P81605|DCD_HUMAN	Dermcidin OS=Homo sapiens GN=DCD PE=1 SV=2	11	55.45
sp|P04196|HRG_HUMAN	Histidine-rich glycoprotein OS=Homo sapiens GN=HRG PE=1 SV=1	9	19.43
sp|P02787|TRFE_HUMAN	Serotransferrin OS=Homo sapiens GN=TF PE=1 SV=3	6	9.74
sp|P01857|IGHG1_HUMAN	Ig gamma-1 chain C region OS=Homo sapiens GN=IGHG1 PE=1 SV=1	5	18.79
sp|P01859|IGHG2_HUMAN	Ig gamma-2 chain C region OS=Homo sapiens GN=IGHG2 PE=1 SV=2	5	17.48
sp|P01861|IGHG4_HUMAN	Ig gamma-4 chain C region OS=Homo sapiens GN=IGHG4 PE=1 SV=1	5	17.43
sp|P19320|VCAM1_HUMAN	Vascular cell adhesion protein 1 OS=Homo sapiens GN=VCAM1 PE=1 SV=1	5	7.85
sp|P12273|PIP_HUMAN	Prolactin-inducible protein OS=Homo sapiens GN=PIP PE=1 SV=1	4	27.40
sp|P02656|APOC3_HUMAN	Apolipoprotein C-III OS=Homo sapiens GN=APOC3 PE=1 SV=1	3	44.44
sp|Q9NZT1|CALL5_HUMAN	Calmodulin-like protein 5 OS=Homo sapiens GN=CALML5 PE=1 SV=2	3	28.77
sp|P01860|IGHG3_HUMAN	Ig gamma-3 chain C region OS=Homo sapiens GN=IGHG3 PE=1 SV=2	3	7.69
sp|P01834|IGKC_HUMAN	Ig kappa chain C region OS=Homo sapiens GN=IGKC PE=1 SV=1	3	48.11
sp|P06702|S10A9_HUMAN	Protein S100-A9 OS=Homo sapiens GN=S100A9 PE=1 SV=1	3	30.70
sp|P02671|FIBA_HUMAN	Fibrinogen alpha chain OS=Homo sapiens GN=FGA PE=1 SV=2	2	2.77
sp|P00738|HPT_HUMAN	Haptoglobin OS=Homo sapiens GN=HP PE=1 SV=1	2	4.68
sp|P0CG04|LAC1_HUMAN	Ig lambda-1 chain C regions OS=Homo sapiens GN=IGLC1 PE=1 SV=1	2	28.30
sp|P0CG05|LAC2_HUMAN	Ig lambda-2 chain C regions OS=Homo sapiens GN=IGLC2 PE=1 SV=1	2	28.30
sp|P0CG06|LAC3_HUMAN	Ig lambda-3 chain C regions OS=Homo sapiens GN=IGLC3 PE=1 SV=1	2	28.30
sp|P0CF74|LAC6_HUMAN	Ig lambda-6 chain C region OS=Homo sapiens GN=IGLC6 PE=4 SV=1	2	28.30
sp|B9A064|IGLL5_HUMAN	Immunoglobulin lambda-like polypeptide 5 OS=Homo sapiens GN=IGLL5 PE=2 SV=2	2	14.02
sp|P01042|KNG1_HUMAN	Kininogen-1 OS=Homo sapiens GN=KNG1 PE=1 SV=2	2	4.35
sp|Q96DR8|MUCL1_HUMAN	Mucin-like protein 1 OS=Homo sapiens GN=MUCL1 PE=1 SV=1	2	13.33

Supplementary Table S6.

Accession	Name	Peptides(95%)	%Cov(95)
sp|P02768|ALBU_HUMAN	Serum albumin OS=Homo sapiens GN=ALB PE=1 SV=2	13	12.81
sp|P02656|APOC3_HUMAN	Apolipoprotein C-III OS=Homo sapiens GN=APOC3 PE=1 SV=1	8	37.37
sp|Q9NZT1|CALL5_HUMAN	Calmodulin-like protein 5 OS=Homo sapiens GN=CALML5 PE=1 SV=2	7	51.37
sp|P81605|DCD_HUMAN	Dermcidin OS=Homo sapiens GN=DCD PE=1 SV=2	5	25.45
sp|P02671|FIBA_HUMAN	Fibrinogen alpha chain OS=Homo sapiens GN=FGA PE=1 SV=2	5	7.16
sp|P10909|CLUS_HUMAN	Clusterin OS=Homo sapiens GN=CLU PE=1 SV=1	4	14.03
sp|P06702|S10A9_HUMAN	Protein S100-A9 OS=Homo sapiens GN=S100A9 PE=1 SV=1	3	24.56
sp|P12273|PIP_HUMAN	Prolactin-inducible protein OS=Homo sapiens GN=PIP PE=1 SV=1	2	15.07
sp|Q8NHM4|TRY6_HUMAN	Putative trypsin-6 OS=Homo sapiens GN=PRSS3P2 PE=5 SV=2	2	8.50
sp|P07477|TRY1_HUMAN	Trypsin-1 OS=Homo sapiens GN=PRSS1 PE=1 SV=1	2	8.50
sp|P07478|TRY2_HUMAN	Trypsin-2 OS=Homo sapiens GN=PRSS2 PE=1 SV=1	2	8.50
